# TNFα-Induced Apoptosis Enabled by CCN1/CYR61: Pathways of Reactive Oxygen Species Generation and Cytochrome c Release

**DOI:** 10.1371/journal.pone.0031303

**Published:** 2012-02-17

**Authors:** Vladislava Juric, Chih-Chiun Chen, Lester F. Lau

**Affiliations:** Department of Biochemistry and Molecular Genetics, University of Illinois College of Medicine, Chicago, Illinois, United States of America; Wayne State University, United States of America

## Abstract

Although TNFα is a strong inducer of apoptosis, its cytotoxicity in most normal cells *in vitro* requires blockade of NFκB signaling or inhibition of *de novo* protein synthesis, typically by the addition of cycloheximide. However, several members of CCN (CYR61/CTGF/NOV) family of extracellular matrix proteins enable TNFα-dependent apoptosis *in vitro* without inhibiting NFκB or *de novo* protein synthesis, and CCN1 (CYR61) is essential for optimal TNFα cytotoxicity *in vivo*. Previous studies showed that CCN1 unmasks the cytotoxicity of TNFα by binding integrins α_v_β_5_, α_6_β_1_, and the cell surface heparan sulfate proteoglycan syndecan 4 to induce the accumulation of a high level of reactive oxygen species (ROS), leading to a biphasic activation of JNK necessary for apoptosis. Here we show for the first time that CCN1 interacts with the low density lipoprotein receptor-related protein 1 (LRP1) in a protein complex, and that binding to LRP1 is critical for CCN1-induced ROS generation and apoptotic synergism with TNFα. We also found that neutral sphingomyelinase 1 (nSMase1), which contributes to CCN1-induced ROS generation, is required for CCN1/TNFα-induced apoptosis. Furthermore, CCN1 promotes the activation of p53 and p38 MAPK, which mediate enhanced cytochrome c release to amplify the cytotoxicity of TNFα. By contrast, LRP1, nSMase1, p53, and p38 MAPK are not required when TNFα-dependent apoptosis is facilitated by the presence of cycloheximide, indicating that they function in the CCN1 signaling pathway that converges with TNFα-induced signaling events. Since CCN1/CYR61 is a physiological regulator of TNFα cytotoxicity at least in some contexts, these findings may reveal important mediators of TNFα-induced apoptosis *in vivo* and identify potential therapeutic targets for thwarting TNFα-dependent tissue damage.

## Introduction

Apoptosis is an important cellular process in embryonic development, immune system function, tissue homeostasis, and tumor suppression. Among the many physiological factors that can trigger apoptosis is tumor necrosis factor α (TNFα), which plays an important role in regulating the immune response [1]. TNFα-induced apoptosis has been implicated in a variety of pathologies linked to chronic inflammation and auto-immune diseases, most demonstrably in liver diseases including alcoholic and inflammatory hepatitis [2], [3], ischemia/reperfusion liver injury [4], and fulminant hepatic disease [5].

TNFα-induced apoptosis is mediated through its cell surface receptor TNFR1 and involves the assembly of two signaling complexes that sequentially activate NFκB and caspases [6]. Binding of TNFα to TNFR1 triggers receptor trimerization and the recruitment of TRADD, RIP1 and TRAF2. This receptor-associated complex promotes the activation of NFκB, a transcription factor that induces expression of many pro-inflammatory, pro-mitogenic, and anti-apoptotic genes [7]. Following TNFR1 endocytosis, TRADD, RIP1 and TRAF2 become modified and dissociate from the receptor in the cytosol, whereupon they bind to FADD to recruit and activate caspases-8/10, leading to apoptosis [6]. However, apoptosis is continuously inhibited by many anti-apoptotic factors whose expression is induced by NFκB. Among these are c-FLIP, which binds FADD and inhibit caspases 8/10 activation [8]; MAPK phosphatases, which can dephosphorylate and inactivate JNK [9]; anti-oxidant proteins such as Mn^++^-SOD and ferritin heavy chain, which inhibit the accumulation of ROS [10]; as well as caspase inhibitors (XIAP, c-IAP1, cIAP2, survivin) and anti-apoptotic members of the Bcl family (Bcl-_XL_, Nr13, and A1/Bfl1) [7], [11]. Therefore, manifestation of TNFα-induced apoptosis in normal cells *in vitro* often requires blockade of NFκB signaling or inhibition of *de novo* protein synthesis, typically by the addition of inhibitors of transcription (e.g., actinomycin D) or protein synthesis such as cycloheximide (CHX) [1].

The ability of TNFα to induce apoptosis *in vivo* may depend on regulation by other factors in the tissue microenvironment, and is therefore context-dependent. Recent studies have shown that CCN1, CCN2, and CCN3, members of the CCN (CYR61/CTGF/NOV) family [12] of extracellular matrix (ECM) proteins, can enable TNFα to induce apoptosis without inhibiting NFκB signaling or *de novo* protein synthesis [13], suggesting that the ECM can profoundly influence the biological response to TNFα. Moreover, knockin mice that express an apoptosis-defective CCN1 mutant are substantially resistant to TNFα-mediated apoptosis *in vivo*, indicating that CCN1 is a physiologic regulator of TNFα cytotoxicity [14]. These findings suggest that CCN1/TNFα-induced apoptosis, which may occur through signaling pathways distinct from one facilitated by the presence of cycloheximide, may more accurately reflect physiological apoptotic processes in certain biological contexts.

A member of the CCN family of matricellular proteins [12], [15], CCN1 (CYR61) regulates diverse cellular responses including cell adhesion, migration, proliferation, differentiation, and survival [16]. Previous studies showed that CCN1 enables TNFα-induced apoptosis by binding integrins α_v_β_5_, α_6_β_1_, and the cell surface heparan sulfate proteoglycan (HSPG) syndecan 4 to trigger the production of reactive oxygen species (ROS) through 5-lipoxygenase and mitochondria, leading to the biphasic activation of JNK critical for apoptosis [14]. Here we have uncovered several additional essential players in CCN1/TNFα-induced apoptosis. First, we show for the first time that CCN1 interacts with the low density lipoprotein receptor-related protein 1 (LRP1) in a protein complex. This interaction with LRP1 contributes to CCN1-induced ROS accumulation and CCN1/TNFα-induced apoptosis, suggesting that LRP1 is a coreceptor for CCN1 critical for mediating these processes. Second, we show that neutral sphingomyelinase 1 (nSMase1), previously shown to participate in the generation of CCN1-induced ROS [17], also contributes to TNFα cytotoxicity. Third, we found that p53 and p38 MAPK are required for facilitating cytochrome c release critical for apoptosis. By contrast, LRP1, nSMase1, p53, and p38 are not required for TNFα-induced apoptosis facilitated by CHX. These findings provide new insights into the signaling pathway for CCN1/TNFα-mediated apoptosis, which operates *in vivo*, and underscore potential targets for intervention in combating TNFα-dependent tissue damage in certain inflammatory diseases.

## Results and Discussion

### LRP1 is a CCN1 coreceptor required for CCN1-induced ROS production and CCN1/TNFα-induced apoptosis

**Figure 1:**
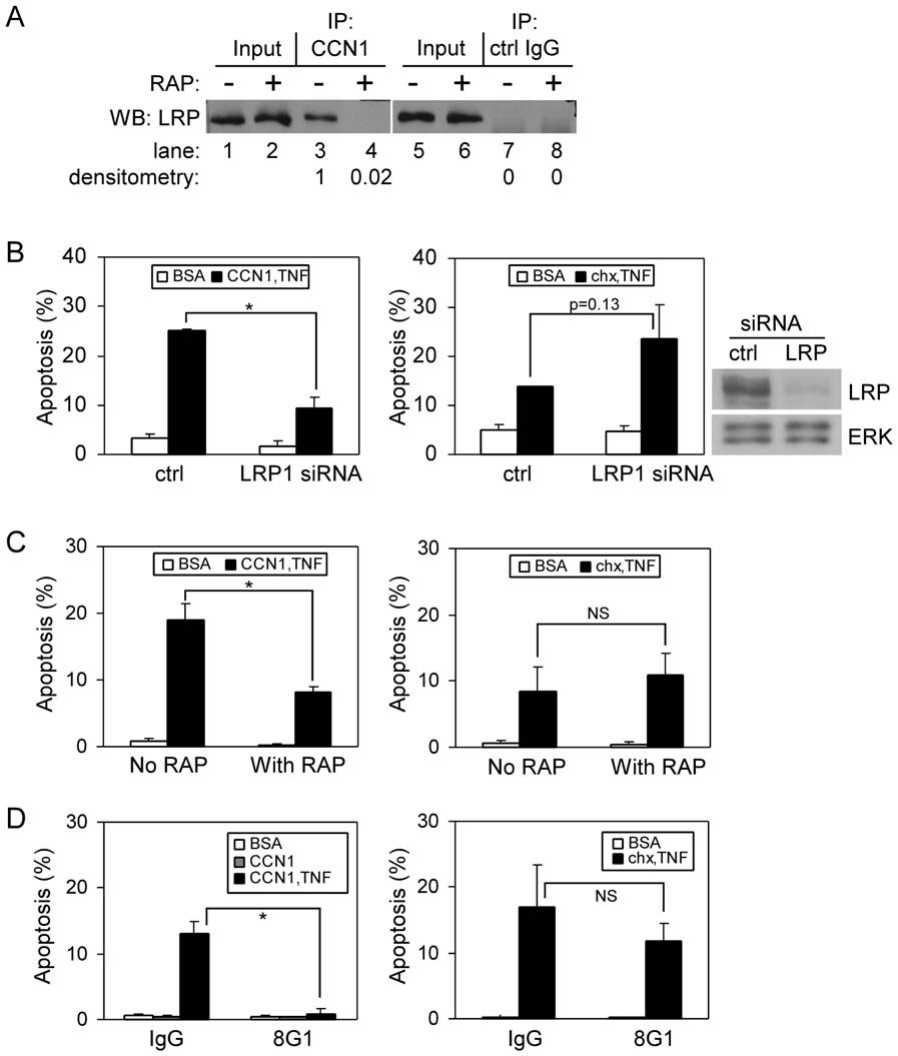
LRP1 is essential for CCN1/TNFα-, but not CHX/TNFα-induced apoptosis. *A.* Detection of LRP1 protein levels in 15 µg of whole cell protein lysates (lanes 1, 2, 5, and 6), representing 3% of lysate used for immunoprecipitation with 7 µg/ml rabbit polyclonal anti-CCN1 antibody (lanes 3 and 4) or control IgG (lanes 7 and 8). Where indicated, 1 µM recombinant RAP was added to the medium and incubated with cells for 30 min before cell lysis. Whole cell protein lysates and immune complexes were resolved on 7.5% SDS-PAGE, immunoblotted, and 515 kDa subunit of LRP1 was detected with monoclonal anti-LRP1. *B.* HSFs were transfected with 80 nM non-targeting (ctrl) or LRP1 siRNA, and cell death was induced after 72 hrs by treatment of cells with CCN1 (2 µg/ml) and TNFα (10 ng/ml) for 5 hrs (left panel; *p<0.01; n = 3), or with CHX (1 µg/ml) and TNFα for 16 hrs (right panel; p = 0.13; NS-not significant, n = 3). Silencing of LRP1 was validated by immunoblot detection of the 515 kDa LRP1 subunit in control or LRP1 siRNA-treated HSFs. ERK1/2 detection serves as a loading control. *C.* Apoptosis in serum-starved HSFs was induced as described, in the presence or in the absence of 1 µM recombinant RAP (left panel: *p<0.01; n = 3; right panel: NS-not significant, n = 3). *D.* HSFs were pre-incubated with 50 µg/ml of isotype control IgG or function-blocking monoclonal antibody against LRP1, 8G1 clone (left panel: *p<0.01; n = 3; right panel: NS-not significant, n = 3).

Low density lipoprotein receptor-related protein 1 (LRP1) is a cell surface endocytic receptor of the low density lipoprotein receptor family [18]. It consists of an extracellular 515 kDa subunit that binds more than 30 diverse ligands and a transmembrane 85 kDa subunit that interacts with several adaptor proteins to mediate endocytosis and signaling. LRP1 is associated with and serves as a coreceptor for many growth factor receptors and integrins, facilitating receptor endocytosis and signaling. LRP1 binds CCN2 [19], a CCN family member homologous to CCN1, and is required for CCN2-mediated cell adhesion and CCN2/TGF-β-induced myofibroblast differentiation [20], [21]. However, its interaction with CCN1 has not been assessed heretofore. In primary human skin fibroblasts (HSFs), we detected the 515 kDa subunit of LRP1 in the protein complex immunoprecipitated with anti-CCN1 antibodies (Fig. 1A, lane 3), but not with isotype control IgG (Fig. 1A, lane7). To establish the interaction between LRP1 and CCN1 further, we tested the effects of recombinant receptor-associated protein (RAP), a chaperone of LRP1 commonly used to inhibit LRP1 ligand binding *in vitro* [22], [23]. Pre-incubation of cells with 1 µM RAP abolished co-immunoprecipitation of LRP1 with CCN1 (Fig. 1A, lane 4). These results indicate that CCN1 physically interacts with LRP1 in a protein complex.

Since LRP1 functions as a coreceptor with integrins for CCN2 [20], [24], [25], we tested the possibility that it may also serve as a coreceptor for CCN1 to mediate apoptosis with TNFα using three approaches. First, we used siRNA to silence LRP1 expression in human skin fibroblasts (HSFs), as shown by immunoblot analysis (Fig. 1B). Strikingly, LRP1 silencing reduced CCN1/TNFα-induced apoptosis by ∼70% compared to control siRNA (Fig. 1B). In contrast, LRP1 siRNA caused an increase, albeit not statistically significant, in TNFα-induced apoptosis facilitated by CHX (Fig. 1B). Second, pre-incubation of HSFs with recombinant RAP inhibited the apoptotic response to CCN1/TNFα by ∼60%, but did not affect cell sensitivity to CHX/TNFα (Fig. 1C). Third, pre-treatment of HSFs with the monoclonal antibody against the LRP1 ectodomain (clone 8G1), but not with control antibody, abrogated CCN1/TNFα apoptosis without affecting CHX/TNFα-induced cell death (Fig. 1D). These results show that LRP1 function is critical for CCN1/TNFα-, but not for CHX/TNFα-induced cytotoxicity.

**Figure 2:**
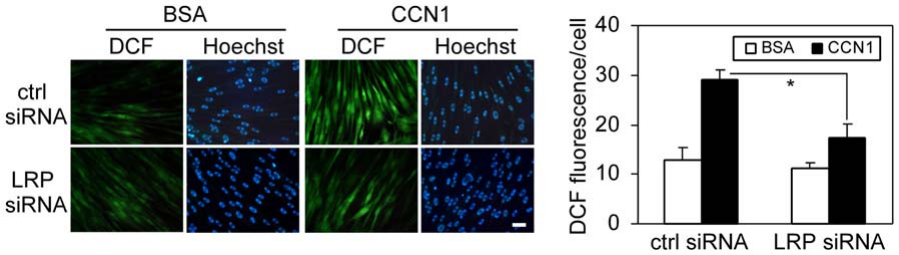
LRP1 mediates CCN1-induced ROS production. *A.* HSFs transfected with 80 nM non-targeting control siRNA or siRNA against LRP1, were used 72 hrs post-transfection for measurement of CCN1-induced ROS by fluorescence microscopy. Cells were loaded with 10 µM CM-H_2_DCF-DA and treated with (2 µg/ml) CCN1 for 15 mins. After nuclear counterstaining with Hoechst 33342 dye, multiple areas of the wells were photographed (left panel) and green fluorescence (DCF) was quantified as described in materials and methods (right panel: *p<0.05, n = 3).

We have previously shown that CCN1 binding to integrins α_v_β_5_ and α_6_β_1_, as well as the HSPG syndecan-4, are required for the generation of a high level of ROS [14]. Inhibition of CCN1-induced ROS by scavengers, or blockade of ROS-generating pathways, annihilated apoptotic synergism between CCN1 and TNFα [14]. Our finding that LRP1 is required for CCN1/TNFα-induced apoptosis prompted us to test whether it plays a role in CCN1-induced ROS production. LRP1 siRNA had no effect on basal ROS level of cells, but reduced CCN1-stimulated ROS levels for ∼50%, as shown by DCF fluorescence (Fig. 2), suggesting that LRP1 is important for optimal ROS production upon CCN1 stimulation and this may explain its role in CCN1/TNFα-induced apoptosis. The partial inhibition of ROS by LRP1 siRNA suggests that CCN1 binding to integrins α_v_β_5_ and α_6_β_1_, and syndecan-4 may account for the remaining CCN1-induced ROS generation.

Taken together, these results indicate that LRP1 serves as a CCN1 coreceptor and contributes to mediating CCN1-induced ROS accumulation, which is critical for apoptotic synergism with TNFα. Since LRP1 can promote endocytosis of many receptor-ligand complexes [26], it is possible that LRP1 is complexed with, and promotes endocytosis of, integrins α_v_β_5_ and/or α_6_β_1_-HSPG upon their interaction with CCN1. Since LRP1 is not required for CHX/TNFα-induced apoptosis (Fig. 1B–D), it appears that LRP1 is not implicated in the internalization of the TNFα receptor 1 (TNFR1), even though TNFR1 internalization is important for TNFα cytotoxicity [27]. The requirement of multiple CCN1 receptors for CCN1/TNFα-induced apoptosis, including LRP1, integrins, and syndecan 4, may contribute to the specificity of the target cells, which must express the correct combination of receptors for responsiveness. Interestingly, a combination of CCN1 mutants that are unable to bind either α_v_β_5_ or α_6_β_1_-HSPG, each defective for apoptosis with TNFα, can fully reconstitute wild type activity [14]. These results indicate that CCN1 can bind the integrins separately and signaling through these receptors converges within the cell to initiate the biological response.

### ROS-dependent p38 MAPK activation is required for CCN1/TNFα-induced apoptosis

**Figure 3:**
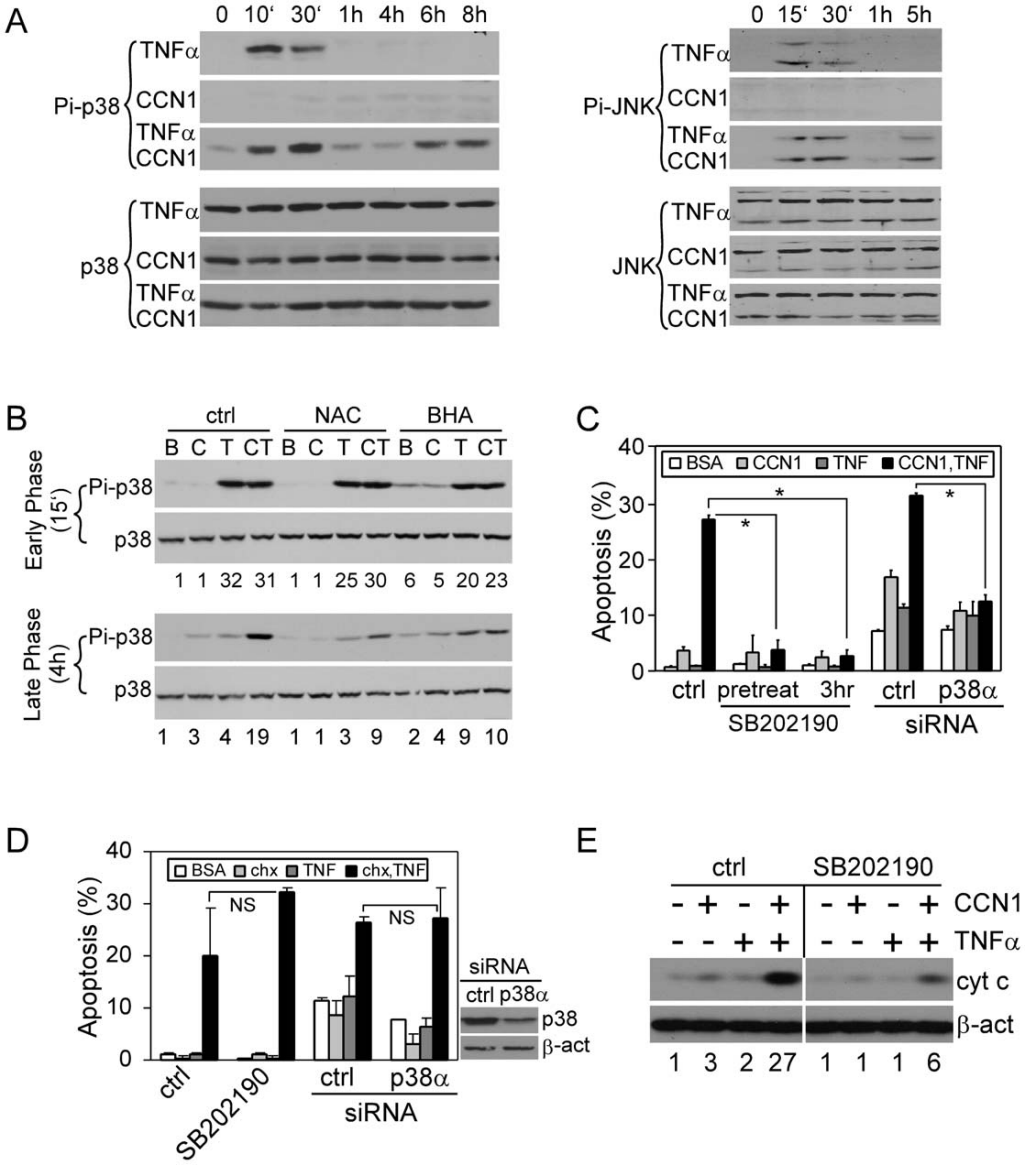
ROS-dependent activation of p38 MAPK is required for CCN1/TNFα-, but not CHX/TNFα-induced apoptosis. *A.* Immunoblot analysis of p38 MAPK (left panel) and JNK activation (right panel) at various times after treatment of cells with CCN1 and/or TNFα. Whole cell lysates were electrophoresed and probed with antibodies against phospho-p38 MAPK (Thr-180/Tyr-182), phospho-JNK1/2 (Thr183/Tyr185), p38 MAPK, and JNK. *B*, Cells were incubated for 30 mins with 10 mM NAC or 5 mM BHA prior to treatment with CCN1 (C) and/or TNFα (T), or BSA (B). Cell lysates collected at early phase (15 mins) or late phase (4 hrs) after CCN1 and/or TNFα treatment were analyzed for phospho-p38 MAPK (Thr-180/Tyr-182) and p38 MAPK levels by immunoblot. Numbers under the immunoblots are relative signal intensities as determined by densitometry. *C.* HSFs were serum-starved overnight and treated with 0.1% DMSO (ctrl) or 20 µM SB202190 for 30 mins, or were subjected to siRNA-mediated silencing of p38α expression. Apoptosis was then induced by treatment of cells with CCN1 (2 µg/ml) and/or TNFα (10 ng/ml) for 5 hrs. Where indicated, SB202190 was added 3 hrs after treatment with CCN1 and TNFα. *p<0.05; n = 3. *D*, Cells were treated with p38 MAPK inhibitor or siRNA as in *C*, and apoptosis was induced with CHX (1 µg/ml) and/or TNFα for 16 hrs (left panel: NS-not significant, n = 3). Silencing of p38α expression was confirmed by immunoblot using antibody against p38 MAPK; β-actin served as control (right panel). *E*, Cells were treated with CCN1 and/or TNFα for 5 hrs, and cytochrome c and β-actin in cytosolic extracts were detected by immunoblotting. SB202190 (20 µM) or 0.1% DMSO (ctrl) were incubated with cells 30 min prior to treatments. Numbers under the immunoblots are relative cytochrome c signal intensities as determined by densitometry.

Elevated ROS levels elicit multiple cellular responses, including the activation of stress response kinases such as p38 MAPK and JNK. This activation is achieved via two mechanisms: 1, ROS can activate ASK1, a redox-sensitive kinase upstream of p38 and JNK [28], and 2, ROS can inactivate MAPK phosphatases by oxidizing the cysteine residues at their active sites, thus preventing the dephosphorylation and inactivation of MAPKs [29]. We showed that CCN1-induced ROS enables a prolonged activation of JNK by TNFα, and that this is critical for CCN1/TNF apoptosis. Here, we tested if CCN1 promotes activation of p38 MAPK in a fashion similar to JNK. We found that TNFα alone, but not CCN1, stimulated the activation of both p38 MAPK and JNK by phosphorylation within 15–30 min., whereas stimulation of cells with both TNFα and CCN1 induced a second phase of p38 MAPK and JNK activation >4 hrs after stimulation (Fig. 3A). The second phase activation of p38 MAPK is inhibited by either NAC or BHA, two different ROS scavengers, but the early phase activation was unaffected (Fig. 3B), showing that this process is ROS-dependent. Considering that ROS regulates activity of many of the redox-sensitive kinases and phosphatases [30], it is unlikely that ROS inhibits p38 MAPK specifically. Indeed, our results demonstrate that ROS inhibitors block not only the second phase of activation of p38 MAPK (Fig. 3B), but also of JNK [14]. The role of p38 MAPK in TNFα-induced apoptosis has been controversial and appears to be cell type- and context-dependent [31], [32]. To test whether p38 MAPK is required when TNFα-induced apoptosis is enabled by CCN1 or CHX, we used either the chemical inhibitor SB202190 to block its activity or siRNA to silence the expression of p38α, the most abundant p38 MAPK isoform in these cells. Either treatment effectively blocked apoptosis induced by CCN1 and TNFα (Fig. 3C,D right panel), but not CHX and TNFα (Fig. 3D). To evaluate the functional significance of the second phase activation of p38 MAPK in apoptosis, we added the inhibitor SB202190 3 hours after CCN1/TNFα treatment, when the early phase activation has already subsided but the late phase activation has not yet begun (Fig. 3C). This treatment completely blocked apoptosis, indicating that the late phase activation of p38 MAPK is essential for CCN1/TNFα-induced apoptosis. Likewise, the late phase activation of JNK is also required for CCN1/TNFα-induced cell death [14].

p38 MAPK can enhance cytochrome c release by promoting mitochondrial translocation of the pro-apoptotic protein Bax from the cytosol, or by phosphorylating Bcl2 to decrease its anti-apoptotic activity [33]. Consistently, we detected a significantly higher level of cytochrome c release from the mitochondria in cells co-treated with CCN1 and TNFα, and the effect is diminished in the presence of the p38 MAPK inhibitor SB202190 (Fig. 3E). In contrast to the context-dependent role of p38 MAPK, JNK activation appears necessary for TNFα-induced apoptosis generally [29]. Pretreatment of HSFs with the JNK inhibitor SP600125 blocked TNFα-induced apoptosis facilitated by either CCN1 or CHX (Fig. S1).

### p53 mediates CCN1/TNFα-induced apoptosis through late phase activation of JNK and p38 MAPK

**Figure 4:**
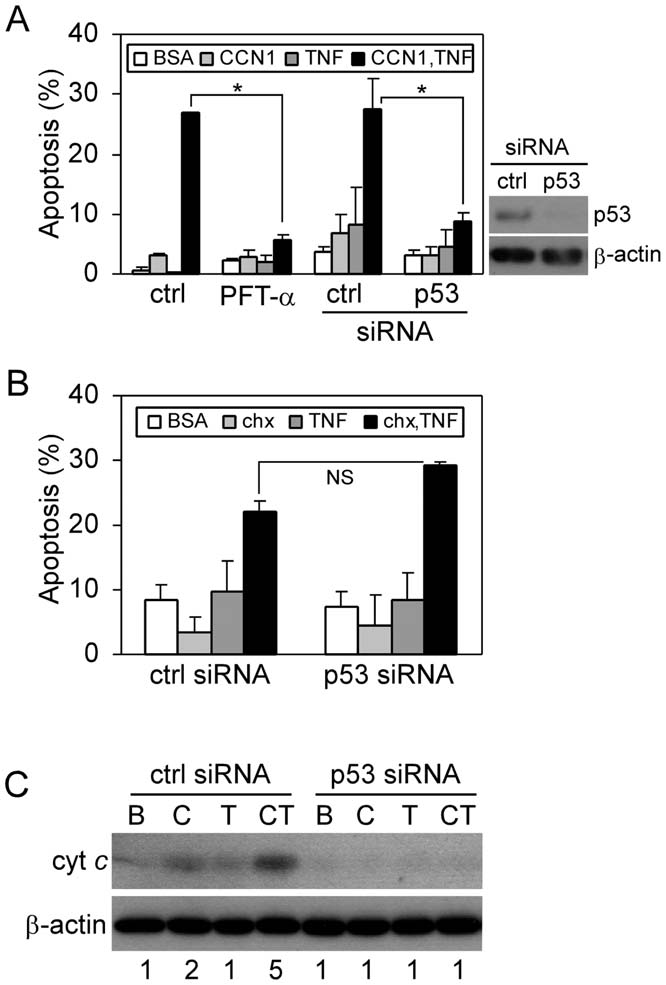
p53 mediates CCN1/TNFα-, but not CHX/TNFα-induced apoptosis. *A.* HSFs were treated with 0.1% DMSO (ctrl) or 100 µM cyclic PFT-α for 30 min, or were subjected to siRNA-mediated silencing of p53 (confirmed by immunoblot using anti-p53 and β-actin as a loading control; right panel). Cells were then stimulated to undergo apoptosis by treatment with CCN1 (2 µg/ml) and/or TNFα (10 ng/ml) for 5 hrs (*p<0.05, n = 3). *B.* Effect of p53 silencing on apoptosis induced by CHX (1 µg/ml) and/or TNFα for 16 hrs was quantified (NS-not significant, n = 3). *C.* Immunoblot detection of cytochrome c in cytosolic extracts of cells treated for 4 hrs with CCN1 (C) and/or TNFα (T), or BSA (B), with and without silencing of p53. Numbers under the immunoblots are relative cytochrome c signal intensities as determined by densitometry.

p53 is a tumor suppressor that functions to promote cell cycle arrest, apoptosis, or senescence in response to various cellular stresses such as DNA damage, hypoxia, or oxidative stress [34]. CCN1 interaction with fibroblasts through integrins and HSPGs leads to a robust and sustained accumulation of ROS, which triggers a DNA damage response that includes the activation of ATM, Chk1, Chk2, and p53 [35]. The activation of p53 downstream of CCN1 prompted us to examine whether p53 is involved in CCN1/TNFα-induced apoptosis. Treatment of cells with the p53 inhibitor cyclic pifithrin-α(PFTα) or siRNA-mediated silencing of p53 completely blocked CCN1/TNFα-induced apoptosis (Fig. 4A), but CHX/TNFα-induced apoptosis was unaffected by p53 silencing (Fig. 4B). Moreover, p53 knockdown blocked cytochrome c release in response to CCN1/TNFα treatment (Fig. 4C). Thus, p53 is critical for mediating TNFα cytotoxicity facilitated by CCN1 but not by CHX, and acts upstream of the mitochondria in this process.

**Figure 5:**
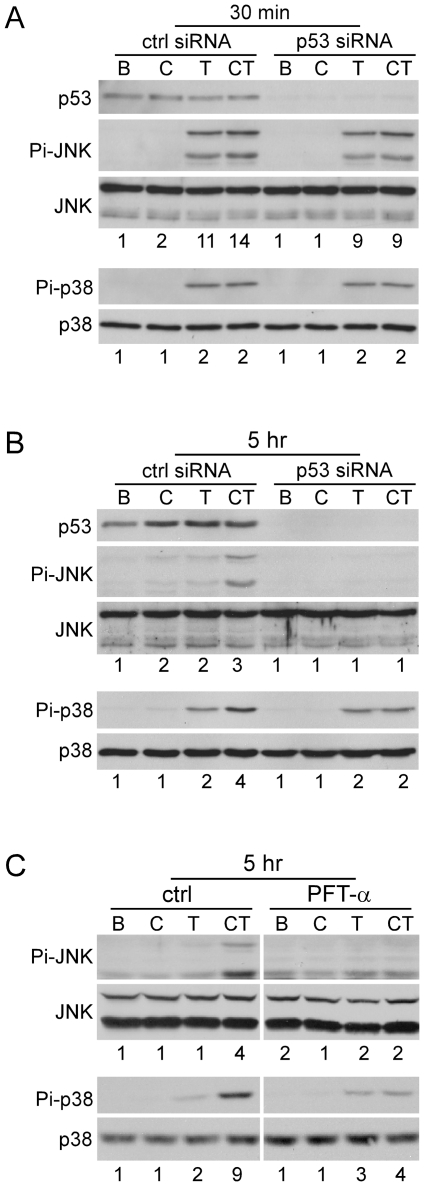
p53 is required for biphasic activation of JNK and p38 MAPK induced by CCN1 and TNFα. *A*, HSFs were transfected with p53 siRNA or control siRNA (50 nM), and treated with BSA (B), CCN1 (C), TNFα (T), or both (CT) for 30 min. Phosphorylation of JNK (Thr183/Tyr185) and p38 MAPK (Thr-180/Tyr-182), and expression of p53 were analyzed by immunoblotting. *B*, cells were transfected as in *A* and phosphorylation of JNK and p38 MAPK was analyzed after 5 hrs of CCN1/TNFα treatment. *C*, serum-deprived HSFs were incubated with 100 µM PFT-α for 30 mins and analyzed after 5 hrs of treatment with proteins as described above.

Next, we monitored the phosphorylation of JNK and p38 MAPK after stimulation by TNFα and CCN1 as a function of p53 expression. Silencing of p53 by siRNA had no effect on the early phase (30 min.) of TNFα-induced JNK and p38 (Fig. 5A), but inhibited the late phase activation (5 hrs) of JNK and p38 by CCN1/TNFα (Fig. 5B), although the low level of p38 activity induced by TNFα alone in the late phase persisted. Similar results were obtained with p53 inhibition by PFT-α (Fig. 5C). These observations indicate that p53 mediates CCN1/TNFα-induced apoptosis at least in part through the activation of JNK and p38 MAPK. Although the late phase phosphorylation of JNK and p38 MAPK is ROS-dependent, p53 is not required for CCN1 to induce ROS accumulation, as chemical inhibition or siRNA silencing of p53 did not diminish CCN1-induced ROS (Fig. S2). These results show that p53 can regulate the activation of p38 MAPK and JNK through an as yet undefined, ROS-independent mechanism.

*In vivo* studies have found that TNFα-induced apoptosis may be p53-dependent [36] or independent [37], possibly as a function of the cellular and tissue context. CCN1/TNFα-induced apoptosis can occur without *de novo* protein synthesis [14], and therefore the role of p53 does not depend upon its transcriptional induction of pro-apoptotic genes such as PUMA, Noxa, Bax, AIP-1, Apaf-1 and PERP. In addition to regulating p38 MAPK and JNK activation (Fig. 5), p53 can promote apoptosis by direct physical interaction with BH3-only proteins such as Bax/Bak, or with Bcl family members to displace BH3-only proteins, thus leading to Bax activation and cytochrome c release [38], [39]. CCN1 alone can induce this mechanism of apoptosis in cells that are deficient in p21 and are thus more susceptible to apoptosis [40].

### nSMase is required for CCN1/TNFα-, but not CHX/TNFα-induced apoptosis

**Figure 6:**
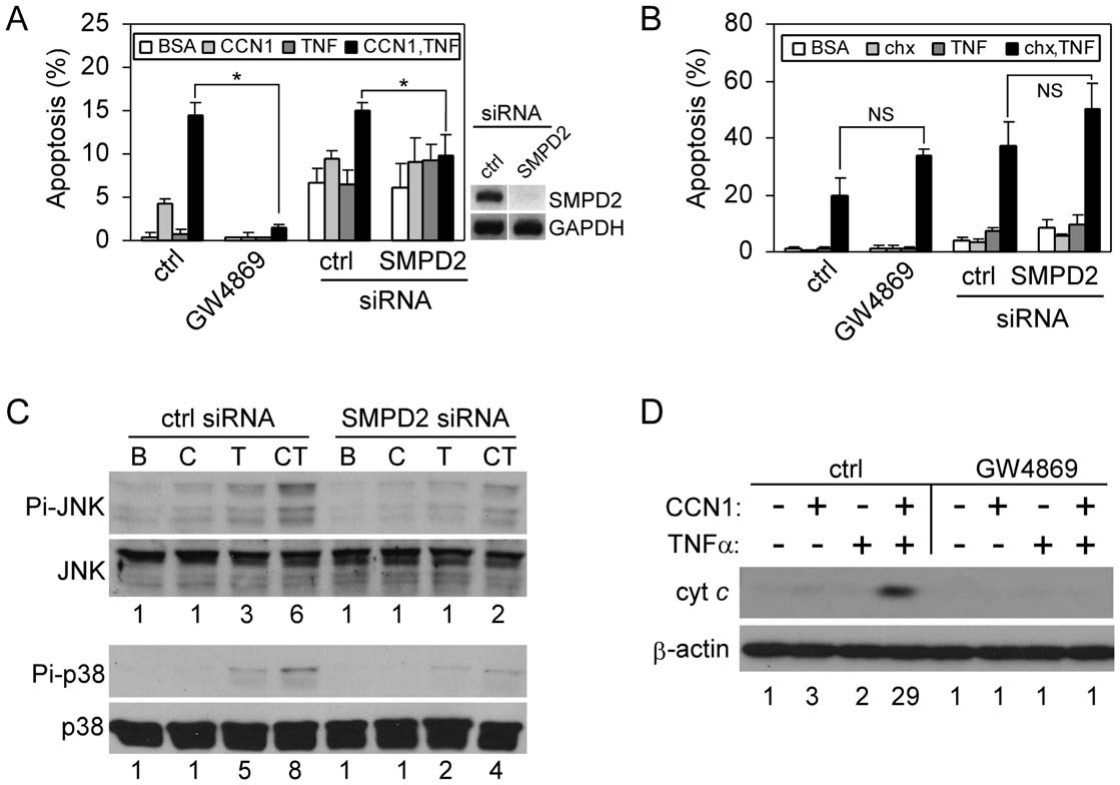
nSMase mediates CCN1/TNFα-, but not CHX/TNFα-induced apoptosis. *A*, HSFs were either treated with 0.1% DMSO (ctrl) or 20 µM GW4869 for 30 mins, or subjected to siRNA-mediated silencing of nSMase1 (SMPD2). Cells were then tested for apoptosis in response to CCN1 (2 µg/ml) and/or TNFα (10 ng/ml). (*p<0.05; n = 3). On the right is RT-PCR analysis of SMPD2 and GAPDH expression in cells transfected with control or SMPD2 siRNA. *B*. Apoptotic response of cells to CHX (1 µg/ml) and/or TNFα was assessed after the inhibition of nSMase with GW4869, or upon siRNA-mediated silencing of nSMase1 (NS-not significant, n = 3). *C*, HSFs were transfected with control siRNA or siRNA against nSMase 1, and stimulated for 5 hrs with BSA (B), CCN1 (C), TNFα (T), or both (CT). Phosphorylation of JNK (Thr183/Tyr185) and p38 MAPK (Thr-180/Tyr-182) was analyzed by immunoblotting. Numbers under the immunoblots are relative signal intensities as determined by densitometry. *D*, HSFs were incubated with GW4869 (20 µM) where indicated, and treated with CCN1 and/or TNFα for 5 hrs. Cytosolic extracts were electrophoresed and cytochrome c was detected by immunoblotting. Numbers under the immunoblots are relative cytochrome c signal intensities as determined by densitometry.

CCN1 synergizes with TNFα to trigger apoptosis in part by inducing ROS generation through 5-lipoxygenase and the mitochondria [14]. Subsequent studies showed that CCN1 also induces ROS accumulation through nSMase1 [41]. Indeed, blockade of nSMase activity with the chemical inhibitor GW4869 abrogated CCN1-induced ROS accumulation, indicating that nSMase plays a critical role in this process (Fig. S3). This requirement of nSMase1 for CCN1-induced ROS prompted us to test whether nSMase1 is involved in TNFα-induced apoptosis. Treatment of cells with GW4869 or silencing of nSMase1 expression by siRNA inhibited CCN1/TNFα-induced apoptosis (Fig. 6A), but did not affect CHX/TNFα-induced apoptosis (Fig. 6B). Silencing of nSMase1 greatly diminished phosphorylation of JNK and p38 MAPK, and inhibition of nSMase with GW4869 efficiently blocked cytochrome c release (Fig. 6C,D). These results indicate that CCN1-induced nSMase1 activity is critical for ROS generation, and contributes to the activation of JNK and p38 MAPK necessary for CCN1/TNFα-induced apoptosis. Inasmuch as nSMase1 knockout mice show no obvious phenotype under normal growth conditions [42], it would be of interest to challenge these mice with inflammatory conditions that invoke TNFα-induced apoptosis, and assess whether their responses are impaired.

TNFα dysregulation is critically involved in pathogenesis of a broad range of chronic and acute inflammatory diseases, and inhibition of TNFα activity has proven an effective therapeutic approach for the treatment of rheumatoid arthritis, psoriasis, and inflammatory bowel disease [43]. Much work has been done to elucidate the molecular mechanism of TNFα-induced apoptosis, although many of these studies were performed either in certain cancer cells carrying mutations that render them responsive to TNFα-induced apoptosis, or in normal cells with the presence of sensitizing agents such as CHX [8], [44]. Like TNFα, CCN1 is highly expressed in response to infections and tissue injury, suggesting that CCN1 can interact with TNFα in many pathological contexts [16]. Indeed, CCN1 is required for TNFα-induced apoptosis in concanavalin A-induced hepatitis and in the skin after direct injection of TNFα, showing CCN1 is a physiological regulator of TNFα-induced apoptosis [14], [45]. Thus, understanding the CCN1/TNFα apoptotic pathway is likely to provide new insight into how TNFα may trigger apoptosis and tissue damage in certain inflammatory diseases.

Upon binding of TNFα to TNFR1, the receptor trimerizes and recruits TRADD, RIP1 and TRAF2 to form a receptor-associated protein complex, which eventually dissociates from the receptor and moves to the cytosol, whereupon it binds to FADD and recruits and activates caspases-8/10, leading to apoptosis [6]. However, TNFα is also a potent activator of NFκB, which inhibits TNFα-induced apoptosis by inducing the synthesis of several anti-apoptotic proteins, including caspase inhibitors, anti-oxidant proteins, MAPK phosphatases, and anti-apoptotic members of the Bcl family [8], [9], [10], [7], [11]. Without affecting NFκB-activation or blocking protein synthesis, CCN1 induces a high level of ROS through nSMase1 [17], 5-LOX, and mitochondria to override the antioxidant effect of NFκB [14], thereby allowing the accumulated ROS to inhibit the NFκB-induced MAPK phosphatases [29] and reactivate JNK and p38 MAPK[14] (Fig. 3A,B). Sustained JNK activation leads to the degradation of c-FLIP [8], whereas re-activated p38 MAPK promotes cytochrome c release (Fig. 3E).

**Figure 7:**
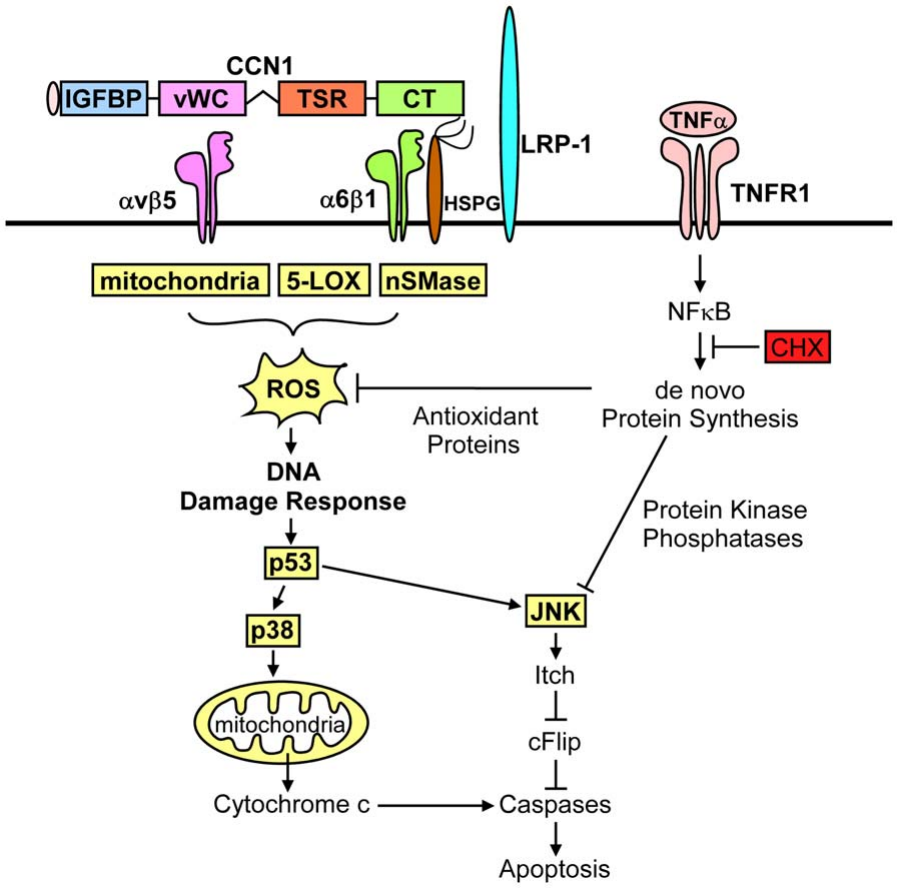
TNFα-induced apoptotic signaling. TNFα acts via its death domain containing receptor TNFR1 to activate caspase 8/10 and promote apoptosis signaling. However, TNFα also activates NFkB to induce expression of a variety of anti-apoptotic genes, resulting in blockade of TNFα-induced apoptosis. The extracellular matrix protein CCN1 unleashes TNFα apoptosis signaling without inhibiting NFkB. Instead, CCN1 acts via integrins α_v_β_5_ and α_6_β_1_, syndecan-4, and LRP1 to induce a high level of ROS in cells, overriding the anti-oxidant effects of NFκB. CCN1-induced ROS, generated via mitochondria, 5-LOX, and nSMase-dependent mechanism, causes a prolonged activation of stress-activated kinases p38 MAPK and JNK. This hyperactivation of kinases depends on p53, which is activated downstream of ROS. JNK enables caspase activation by TNFα by inducing the degradation of caspase inhibitor cFLIP via ubiquitin ligase Itch. In contrast, CCN1-mediated activation p38 MAPK promotes cytochrome c release to facilitate TNFα-mediated apoptosis at the level of mitochondria. In contrast to CCN1, CHX triggers TNFα-mediated apoptosis by blocking de novo protein synthesis, including the synthesis of NFkB targets, such as anti-oxidant proteins and protein kinase phosphatases. This results in accumulation of ROS, activation of JNK, and apoptosis, without the requirement of p38 MAPK or p53.

As members of the CCN family are emerging as potential therapeutic targets in diseases associated with chronic inflammation [12], a detailed understanding of how CCNs synergize with inflammatory cytokines to induce apoptosis is of critical importance. Here we have identified four previously unknown essential participants in the CCN1/TNFα apoptosis pathway: LRP1, nSMase1, p53 and p38 MAPK (Fig. 7). None of them is necessary when TNFα cytotoxicity is facilitated by CHX, suggesting that their functions may counteract the activity of NFκB-induced anti-apoptotic proteins. These findings indicate that TNFα-induced apoptosis under physiological conditions may proceed in a manner distinct from its occurrence in the presence of CHX. Given the importance of CCN1 in TNFα-induced apoptosis in hepatitis [13], assessing whether these participants in the CCN1/TNFα apoptosis pathway play significant roles in TNFα-induced tissue injuries in inflammatory diseases may help to identify potential targets for therapeutic intervention [12]. Even though anti-TNFα therapy is successful in treating several inflammatory diseases, it is often accompanied by increased bacterial and viral infections [46], [47], and increased risk for the development of lymphomas [48]. Thus, development of novel therapeutics may be useful in overcoming the limitations of anti-TNFα therapy. Targeting of LRP1 or nSMase1, individually or in combination, may selectively block TNFα-mediated apoptosis while preserving other protective functions of this cytokine. Inasmuch as p53 and p38 MAPK play important roles in tumor suppression [49], [50], their inactivation may be an inappropriate therapeutic strategy in some contexts. Therefore, LRP1 and nSMase1 signaling pathways may be promising therapeutic targets in treating conditions related to excessive TNFα-induced apoptosis.

## Materials and Methods

### Cell culture

Data presented in this study were obtained using 1064Sk human skin fibroblasts (HSFs) derived from skin biopsies of a healthy newborn (American Type Culture Collection CRL-2076). We also tested three other primary neonatal fibroblast isolates (BJ, 1077 Sk, HFF-1) for apoptotic synergy between CCN1 and TNFα, and found that they all responded similarly. Fibroblasts were maintained in the Iscove's modified Dulbecco's medium (IMDM; Invitrogen) with 10% fetal bovine serum (FBS, Hyclone) at 37°C and 5% CO_2_, and used before reaching 25 population doublings. Before experiments, cells were serum-deprived by overnight incubation in IMDM containing 0.1% bovine serum albumin (BSA).

### Antibodies and reagents

Human recombinant TNFα was from Axxora. Antibodies for phospho-p38 MAPK (Thr-180/Tyr-182), c-Jun N-terminal protein kinase 1/2 (JNK1/2), and ERK1/2 were from Cell Signaling Technology, for phospho-JNK1/2 (Thr183/Tyr185) from Assay Designs, for p38 MAPK and p53 (DO-1) from Santa Cruz Biotechnology, for cytochrome c from Clontech, and antibody against 515 kDa subunit of LRP1 (8G1) was from Fitzgerald Industries. Anti-β-actin antibody (AC-15), CHX, NAC, BHA, and neutral sphingomyelinase inhibitor GW4869 were from Sigma. Inhibitors of p38 MAPK (SB202190), JNK (SP600125), and p53 (cyclic pifithrin-α and pifithrin-μ) were from Calbiochem. ROS detection dyes 5-(and 6-) chloromethyl-2′,7′-dichlorodihydrofluorescein diacetate (CM-H_2_DCF-DA) and dihydrocalcein acetoxymethylester (DHC-AM) were from Invitrogen.

### Coimmunoprecipitation of LRP1 and CCN1

HSFs were rinsed twice with ice-cold PBS and lysed in immunoprecipitation (IP) buffer (20 mM Tris-HCl, pH 7.5, 150 mM NaCl, 1 mM MgCl_2_, 1 mM CaCl_2_, 1% Triton X-100, and EDTA-free protease inhibitor cocktail). After 5 min of incubation at 37°C, cell lysates were passed through a 22-gauge needle 10 times and cleared by centrifugation at 14,000×g at 4°C for 20 min. Protein concentration in supernatants was adjusted to 1 mg/ml and 0.5 ml of the lysate was combined with 3.5 µg of CCN1 antibody or control purified normal rabbit IgG. After overnight incubation at 4°C, lysate-antibody mixture was combined with 50 µl of 25% slurry Protein A/G PLUS-Agarose (Santa Cruz Biotechnology) and incubated for another 4 hrs at 4°C. The beads were then washed once with IP buffer and twice with 0.1% Triton X-100-containing IP buffer. Bead pellets were combined with 40 µl of 2X non-reducing sodium dodecyl sulfate (SDS) buffer and boiled for 5 min. After centrifugation, the supernatants were separated by 7.5% SDS-PAGE and LRP1 expression was analyzed by Western blotting.

### Expression and purification of recombinant CCN1 and RAP

Human recombinant CCN1 protein was produced in insect cells using a baculovirus expression system [51] GST-RAP fusion protein was expressed in BL21 *E.coli* transformed with pGEX-2T-RAP/TEV (kindly provided by Dr. Klavs Dolmer, University of Illinois at Chicago). RAP was purified as described before [52]. First, GST-RAP was affinity-purified using glutathione sepharose 4B (GE Healthcare). GST-RAP was then digested by overnight incubation with TEV protease (Promega) and the GST tag was removed on GSH-Sepharose. Purity of the recombinant proteins was verified by Commassie staining after SDS-PAGE.

### Apoptosis assays

HSFs were incubated in serum-free medium overnight then treated with recombinant proteins and/or inhibitors to induce apoptosis. Cells were fixed with 10% formalin and stained with DAPI (4′,6-diamidino-2-phenylindole, 1 µg/ml). Apoptotic cells were counted under a fluorescent microscope in 10 randomly chosen high power fields (∼300 cells/sample were counted). Data are presented as mean % apoptosis ± standard deviation (SD) from triplicate samples. All apoptosis assays were done at least three times with similar results.

### Immunobloting

Cell lysates were made in RIPA buffer (50 mM Tris-HCl pH 8.0, 150 mM NaCl, 1% (w/v) NP-40, 0.5% sodium deoxycholate, and 0.1% SDS) and were diluted in Laemmli buffer prior to SDS-PAGE. LRP1 was detected under non-reducing conditions, after combining cell lysates with 2X non-reducing SDS buffer (125 mM Tris-HCl pH 6.8, 2.5% SDS, 25% glycerol and 2 mg/ml N-ethylmaleimide). Immunoblotting and protein detection were done using standard methods. X-ray images were scanned and relative band intensity was measured using ImageJ 1.36b software (NIH).

### Cytochrome c release from mitochondria

Cytosolic extracts free of mitochondria were prepared as described [53]. Briefly, cells were harvested by trypsinization and lysed for 10 min at 25°C in 0.5 ml of mannitol/sucrose buffer (20 mM HEPES-KOH, (pH 7.5), 210 mM sucrose, 70 mM mannitol, 1.5 mM MgCl_2_, 10 mM KCl_2_, EDTA-free protease inhibitor cocktail (Roche), and 20 µg digitonin/million cells). After centrifugation at 14,000×g, supernatants representing cytosolic fractions, containing 50 µg of protein, were analyzed for the presence of cytochrome c and β-actin by immunoblot.

### ROS measurements

Reactive oxygen species in live cells were detected by fluorescence microscopy (Leica, DM IRB), using cell-permeable indicator dyes 5-(and 6-) chloromethyl-2′,7′-dichlorodihydrofluorescein diacetate (CM-H_2_DCF-DA) [54] or DHC-AM [55]. The reduced and acetylated indicator dyes are non-fluorescent, but removal of the acetate groups by cellular esterases and oxidation by peroxides, convert them into green-fluorescent products reflecting the amount of ROS. Cells were loaded with 10 µM ROS indicator dye and treated with CCN1 for 10 mins. After counterstaining with Hoechst 33342, five random high-power fields of each sample were photographed using QImaging Retiga 2000R camera. Green fluorescence intensities were measured with ImageJ 1.36b software (NIH) and expressed as fluorescence intensity per cell. For measurement of ROS by flow cytometry (supplementary data), serum-deprived cells were labeled with CM-H_2_DCF-DA (5 µM) for 15 mins at 37°C in the dark, and stimulated for 10 mins with recombinant CCN1. After rinsing and detachment with trypsin/EDTA, cells were suspended in 5% FBS-containing PBS at the concentration of 2×10^5^ cells/ml. Fluorescence of 10^5^ cells was immediately analyzed by flow cytometry (Cell Lab Quanta SC MPL; Beckman Coulter). Data were expressed as geometric mean fluorescence ± SD from triplicate samples.

### siRNA

Cells were transfected with siRNAs using Lipofectamine 2000 reagent (Invitrogen) according to manufacturer's protocol, and assayed for apoptosis or ROS 72 hrs after transfection. Control non-targeting siRNA and siGENOME SMARTpool siRNA against nSMase1 (50 nM) were from Dharmacon. siRNAs against LRP1 (sense: GCAGUUUGCCUGCAGAGAU-dTdT) [56], p38α (sense: CCUACAGAGAACUGCGGUU-dTdT) [57], and p53 (sense: GACUCCAGUGGUAAUCUAC-dTdT) [58] were from Ambion. The silencing was validated using RT-PCR in case of nSMase1 siRNA treatment, or by immunoblot in case of LRP1, p38, and p53 siRNAs.

### Semiquantitative RT-PCR

Total cellular RNA was extracted and cDNA synthesized using standard methods. PCR primers specific for nSMase1 (forward, 5′-CAACAAGTGTAACGACGATGCC-3′; reverse, 5′-CGATTCTTTGGTCCTGAGGTGT-3′) and GAPDH (forward, 5′-ATCGTGGAAGGACTCATGACCACA-3′; reverse, 5′-CCTGCTTCACCACCTTCTTGATGT-3′) were used.

### Statistical analysis

Data are reported as the mean ± SD, and analyzed using Student's t-test. p values≤0.05 were considered significant.

## Supporting Information

Figure S1 **JNK is required for TNFα cytotoxicity induced both by CCN1 and CHX.** *A*, serum-deprived HSFs were treated with 0.1% DMSO (ctrl) or 15 µM SP600125 for 30 mins prior to apoptosis induction with CCN1 (2 µg/ml) and/or TNFα (10 ng/ml) for 5 hrs, and apoptosis was assayed. *B*, cells were prepared as in *A* but apoptosis was induced with CHX (1 µg/ml) and or TNFα (10 ng/ml) for 16 hrs; *p<0.05; n = 3.
(TIF)

Figure S2 **p53 is not required for CCN1-induced ROS accumulation.** *A*, HSFs were incubated with 0.1% DMSO (ctrl) or 10 µM PFTμ for 30 mins before loading with 5 µM CM-DCFDA. Cells were then treated either with BSA or CCN1 (2 µg/ml) and harvested after 10 mins for measurement of ROS by flow cytometry. *B*, HSFs were transfected with p53 siRNA, or control siRNA, and ROS was detected as above. Results are expressed as geometric mean fluorescence ± SD. *p<0.01; n = 3.
(TIF)

Figure S3 **nSMase1 is required for CCN1-induced ROS accumulation.** Serum-deprived HSFs were loaded with 10 µM DHC-AM and treated with CCN1 for 15 mins. After nuclear counterstaining with DAPI, cells were photographed (left). Green fluorescence (DHC-AM) was quantified (right panel) to reflect ROS levels.
(TIF)
