# Supplementary figures and images for: TNFα-Induced Apoptosis Enabled by CCN1/CYR61: Pathways of Reactive Oxygen Species Generation and Cytochrome c Release
Source: PLoS One. 2012 Feb 17;7(2):e31303. doi: 10.1371/journal.pone.0031303 (PMC3281933; doi:10.1371/journal.pone.0031303)

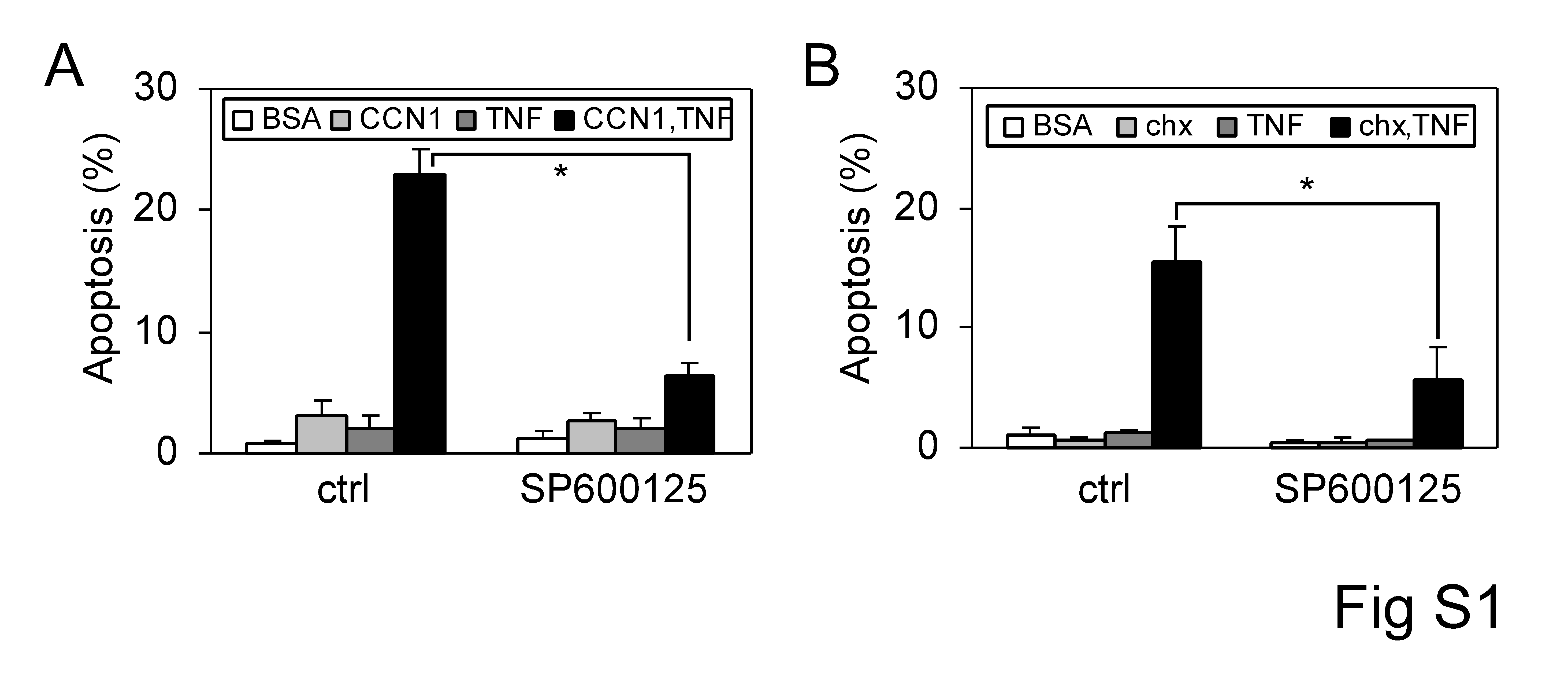

Supplement: Figure S1 — JNK is required for TNFα cytotoxicity induced both by CCN1 and CHX. A, serum-deprived HSFs were treated with 0.1% DMSO (ctrl) or 15 µM SP600125 for 30 mins prior to apoptosis induction with CCN1 (2 µg/ml) and/or TNFα (10 ng/ml) for 5 hrs, and apoptosis was assayed. B, cells were prepared as in A but apoptosis was induced with CHX (1 µg/ml) and or TNFα (10 ng/ml) for 16 hrs; *p<0.05; n = 3. (TIF) [file pone.0031303.s001.tif]

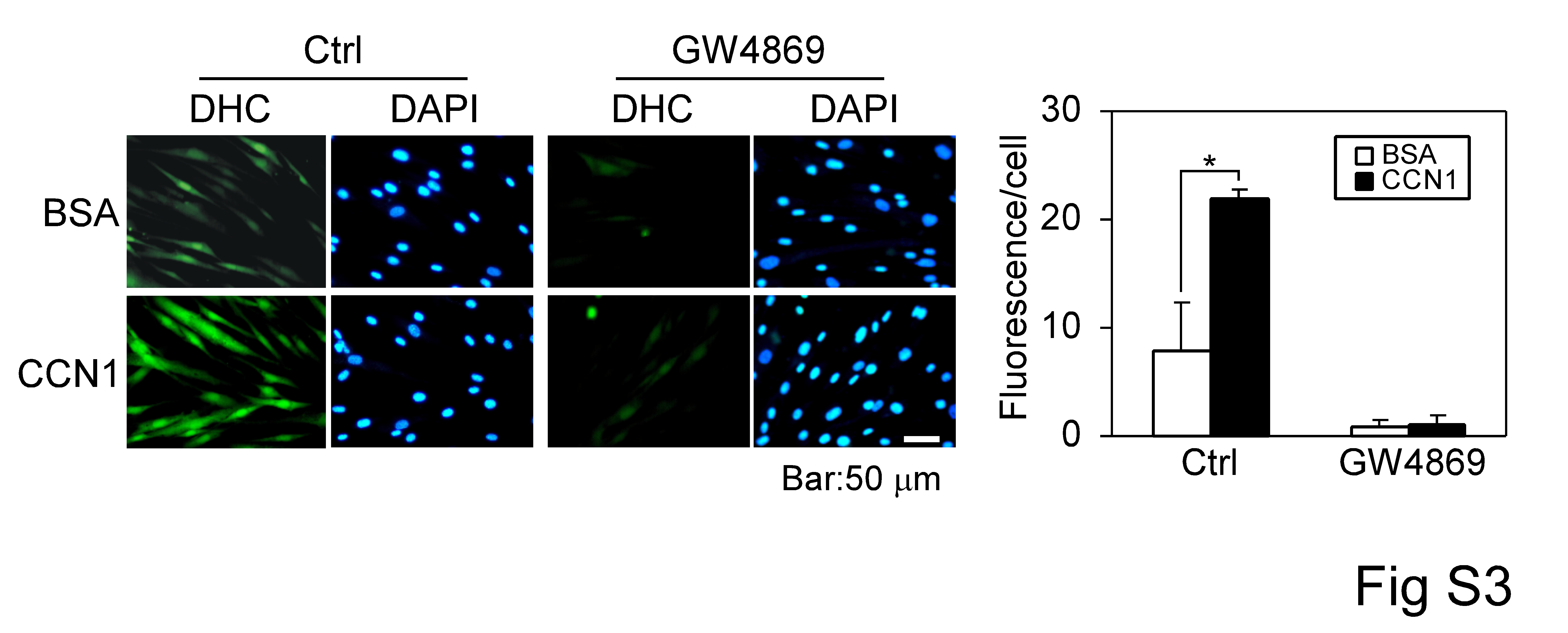

Supplement: Figure S3 — nSMase1 is required for CCN1-induced ROS accumulation. Serum-deprived HSFs were loaded with 10 µM DHC-AM and treated with CCN1 for 15 mins. After nuclear counterstaining with DAPI, cells were photographed (left). Green fluorescence (DHC-AM) was quantified (right panel) to reflect ROS levels. (TIF) [file pone.0031303.s003.tif]
